# Predicting Throughput and Melt Temperature in Pharmaceutical Hot Melt Extrusion

**DOI:** 10.3390/pharmaceutics14091757

**Published:** 2022-08-23

**Authors:** Tobias Gottschalk, Cihangir Özbay, Tim Feuerbach, Markus Thommes

**Affiliations:** 1 Laboratory of Solids Process Engineering, Department of Biochemical and Chemical Engineering, TU Dortmund University, Emil-Figge-Str. 68, 44227 Dortmund, Germany; 2 INVITE GmbH, Drug Delivery Innovation Center, Chempark Building W32, 51368 Leverkusen, Germany

**Keywords:** hot melt extrusion, solid dispersion, process parameter, quality by design, one dimensional, simulation, model

## Abstract

Even though hot melt extrusion (HME) is a commonly applied process in the pharmaceutical area, determination of the optimal process parameters is demanding. The goal of this study was to find a rational approach for predetermining suitable extrusion parameters, with a focus on material temperature and throughput. A two-step optimization procedure, called scale-independent optimization strategy (SIOS), was applied and developed further, including the use of an autogenic extrusion mode. Three different polymers (Plasdone S-630, Soluplus, and Eudragit EPO) were considered, and different optimal process parameters were assessed. The maximum barrel load was dependent on the polymers’ bulk density and the extruder size. The melt temperature was influenced by the screw speed and the rheological behavior of the polymer. The melt viscosity depended mainly on the screw speed and was self-adjusted in the autogenic extrusion. A new approach, called SIOS 2.0, was suggested for calculating the extrusion process parameters (screw speed, melt temperature and throughput) based on the material data and a few extrusion experiments.

## 1. Introduction

A major challenge in today’s pharmaceutical research is the poor solubility of newly developed drugs [1,2]. Even though this has been an issue for several decades, the research in this field is ongoing [3]. Multiple approaches, such as particle size reduction and complex formation, have been considered over the years [4]. One promising approach is the formation of amorphous solid dispersions (ASDs) [5,6]. According to this formulation strategy, the drug substance is dissolved in an amorphous carrier, both to avoid its recrystallizing over time and to maintain the desired dissolution rate in the patient [7,8].

**Figure 1:**
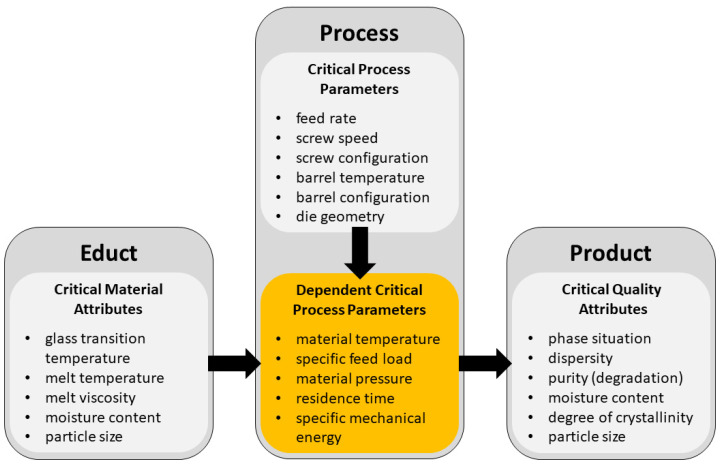
Different types of influencing parameters in hot melt extrusion (modified from [13]).

A common manufacturing technique for ASDs is hot melt extrusion (HME) [5,9]. Corotation twin-screw extruders are frequently used because they have a high mixing capacity and are well known in numerous industries [3,10]. During extrusion, several unit operations (conveying, mixing, melting and softening) take place simultaneously [11,12]. Since those mechanisms are interconnected, the effects cannot be addressed separately [12]. Therefore, even after years of research, not all processes inside the extruder are well understood. An additional challenge is the large number of process parameters influencing the unit operations and the HME in total [3,13]. Those can be divided into groups according to Kolter et al. [13] (see Figure 1). 

The critical process parameters are directly adjustable by the operator. In contrast, the dependent critical process parameters are not directly selectable but are related to the critical process parameters, as well as to the critical material attributes [13]. 

**Figure 2:**
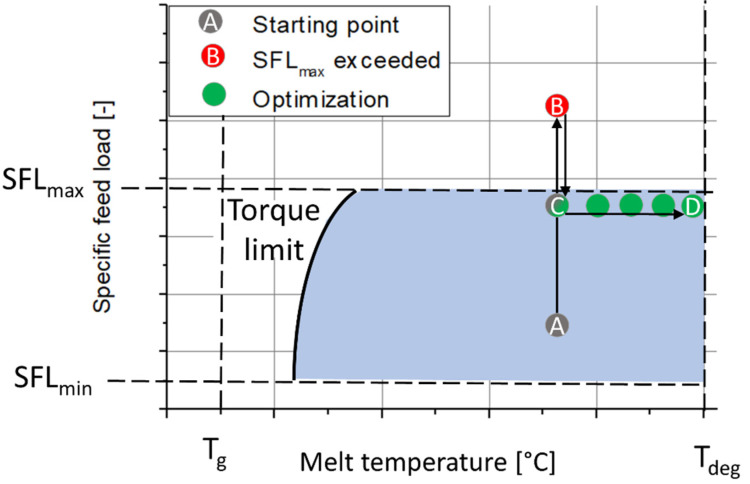
Schematic presentation of the SIOS. The resulting operating window is depicted in blue (according to [14]).

Recently, the scale-independent optimization strategy (SIOS) was proposed as a method to tailor an optimized extrusion process [14]. According to the SIOS, the melt temperature, as well as the specific feed load (SFL) [12,13], are categorized as dependent critical process parameters (see Figure 2). The SFL is a representative, dimensionless number commonly used to describe the barrel load and is calculated from the throughput of the powder ($\dot{m}$), the true density of the polymer ($\rho_{true}$), the screw speed (n) and the screw diameter (d) (Equation (1)) [12].

(1) $$SFL=\frac{\dot{m}/\;\rho_{true}}{n\cdot d^{3}}$$

The SIOS is a two-step efficiency optimization procedure for reaching an optimized operating point, which aims for a high throughput and a low power consumption while producing a homogeneous cylindrical strand (coherent extrudate). During the first step (point A to B), the screw speed is decreased stepwise at a constant throughput until the barrel is completely filled (point B). This point can be detected from a developed backlog in the feeding zone. Thereafter, a slightly higher screw speed is used (point C). In the second step (point C to D), the throughput and the screw speed are increased simultaneously to increase the efficiency of the process at a constant barrel load until degradation is observed (point D). 

Operating the extruder without external barrel heating or cooling is often called autogenic or adiabatic extrusion. It is used to increase the robustness and scalability of the process, since the surface-to-volume ratio of the process unit (barrel and screw) becomes irrelevant [13]. These terms are used interchangeably throughout the literature, but they are not the same, as explained by the energy balance in Equation (2) [13,15].

(2) $$P_{motor}+{\dot{Q}}_{heating}={\dot{Q}}_{temp}+{\dot{Q}}_{loss}+P_{pressure}$$

The motor power ($P_{motor}$) and the heating of the barrel temperature control (${\dot{Q}}_{heating}$) are the power sources in the HME. The temperature elevation of the material (${\dot{Q}}_{temp}$), the power loss to the environment (${\dot{Q}}_{loss}$) and the power consumption for the pressure build-up ($P_{pressure}$) are the power sinks. In autogenic extrusion, the energy input (and output) via barrel temperature control is set to zero by turning off the barrel temperature control (${\dot{Q}}_{heating\;}=0$). Therefore, the power input via screw rotation must be sufficient to keep up with the power consumption [16]. In adiabatic extrusion, the heating, as well as the power loss to the environment, are neglected (${\dot{Q}}_{heating\;}=0\;\mathrm{and}\;{\dot{Q}}_{loss}=0$) [15]. Thus, this can be considered an ideal case, which is not relevant for industrial processes. Since relevant thermal losses to the environment occur through the extruder barrels, this study uses autogenic extrusion. That is, ${\dot{Q}}_{heating\;}=0$, but ${\dot{Q}}_{loss}\neq 0$.

In this work, a theoretical approach was developed for predetermining the optimal process parameters to produce a coherent extrudate at a maximum throughput and defined melt temperature. Therefore, autogenic extrusion was added to the scale-independent optimization strategy. The approach was applied to different polymers, and its applicability to industrial processes was investigated. A mathematical model for the barrel load and the melt temperature was developed, with the aim of predicting the optimized operating point in accordance with the SIOS.

## 2. Materials and Methods

### 2.1. Materials

Three pharmaceutical polymers commonly used in hot melt extrusion were utilized in this study [9,17,18]. These were chosen based on the different rheological behavior of their melts [19,20]: Polyvinylpyrrolidone vinyl acetate (PVPVA) (Plasdone S-630, Ashland Inc., Columbus, OH, USA); the commonly utilized graft copolymer, Soluplus (SOL) (BASF SE, Ludwigshafen, Germany); and the basic butylated methacrylate copolymer (bBMA) (Eudragit EPO, Evonik Industries AG, Darmstadt, Germany). The material densities were determined with a helium pycnometer (Micro UltraPyc 1200E, Quantachrome, Baynton Beach, FL, USA) and bulk densities (untapped densities) were determined using a tapped density tester (Sotax TD1, Sotax, Aesch, Switzerland) in accordance with the European Pharmacopoeia [21].

### 2.2. Hot Melt Extrusion

**Figure 3:**
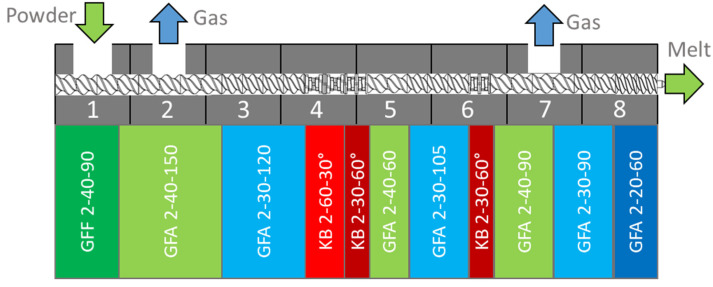
Screw and barrel configuration. Green and blue symbolize conveying elements, red marks kneading zones, the numbers indicate the cylinder elements. Nomenclature according to Leistritz.

A loss-in-weight feeder (K-Tron K-ML-SFS-KT20, Coperion, Niederlenz, Switzerland) was used for dosing the material in the extruder. The extrusion experiments were carried out in a corotating twin-screw extruder (ZSE 27 MAXX, Leistritz, Nuremberg, Germany), containing modular screw elements with a 28.3 mm diameter and a length of 32 D (Figure 3). A heated extrusion die with a 3 mm diameter and 11.7 mm length was utilized and the die pressure was measured using a pressure gauge (KE1-7-M-B35D-1-4-D-S-P-E, Gefran, Provagilo d’Iseo, Italy).

The screw and barrel design was similar to that which was used in previous investigations [14]. For all operating conditions, the melt temperature at the die was measured in triplicate with an IR camera (TESTO 875, Testo SE & Co. KGaA, Lenzkirch, Germany) using the material-specific emission coefficients (PVPVA, SOL, bBMA: 0.93, 0.96, 0.93) when the torque and the pressure at the die had reached a constant value (steady state). For the autogenic extrusion, the measurement was made after reaching a constant barrel temperature.

### 2.3. Scale-Independent Optimization Strategy

For starting the extrusion, a barrel temperature profile was set. Barrels 1 and 2 were cooled to 20 °C and barrel 3 was heated to 80 °C. The subsequent barrel elements were set to the material manufacturers’ recommended temperatures for extruding the excipients (150 °C for PVPVA, 140 °C for SOL and 130 °C for bBMA). During the first optimization step, the screw speed was decreased stepwise (from 200 to 20 rpm) at a constant throughput (3 kg/h). For further processing, the barrel temperature control in barrels 1–3 remained activated to avoid sticking in the feeding zone. Temperature control was disabled in barrels 4–8 to perform autogenic extrusion, which is an extension of the SIOS. In the second optimization step, the throughput was increased stepwise from 3 kg/h up to 42 kg/h. The screw speed was increased accordingly to keep the specific feed load constant (Equation (1)). 

### 2.4. Rheological Investigation

The rheological data for all three polymers were taken from the literature. The datasets were chosen based on five criteria:

1. The temperatures used for rheology measurements were close to the extrusion temperatures.
2. Oscillation rheology measurements covered the high shear rate range relevant for extrusion.
3. Dried polymers were utilized due to the plasticizing effect of the water on the polymers.
4. Sample preparation via “MeltPrep” technology was preferred to minimize the air bubbles in the molten polymer.
5. Repetitive measurements were made to enable confidence in the data.

The literature data were refitted to the Carreau model (Equation (3)) [22] which was coupled with the Arrhenius equation (Equation (4)) [22] to account for different temperatures, since all experiments were far above the glass transition temperature of the polymers [23].

(3) $$\eta =\frac{\eta_{0}a_{T}}{{\left(1+\frac{\dot{\gamma }a_{T}}{{\dot{\gamma }}_{c}}\right)}^{c}}$$

(4) $$a_{T}=e^{\frac{E_{A}}{R}\left(\frac{1}{T}-\frac{1}{T_{Ref}}\right)}$$

The Carreau model describes the dynamic viscosity (η) as a function of the shear rate ($\dot{\gamma }$) using three parameters—namely the viscosity at zero shear rate ($\eta_{0}$), the critical shear rate (${\dot{\gamma }}_{c}$) and the flow index (c). The included shift factor ($a_{T}$) links Carreau to Arrhenius using the time–temperature–superposition approach [12]. The temperature-dependent shift factor is calculated based on the ideal gas constant (*R*) and the temperature (*T*). A material-specific activation energy ($E_{A}$) and a reference temperature ($T_{Ref}$) are used as well (Table 1).

## 3. Results and Discussion

### 3.1. SIOS for Different Polymers

**Figure 4:**
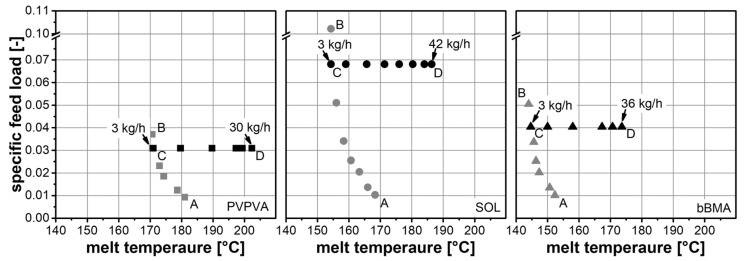
Results of the extrusion experiments. SIOS plots for PVPVA (square), SOL (circle) and bBMA (triangle). Determination of maximum specific feed load (gray) and determination of maximum throughput (black).

The process conditions in extrusion were chosen in accordance with the SIOS [14]. In this two-phase optimization procedure, the maximum specific feed load was determined first (Figure 4, A to B, Table A1) by lowering the screw speed at a constant feed rate (3 kg/h). The minimum screw speed, which leads to an extrusion process without any backlog in the feeding section (Figure 4, C), is used to calculate the maximum specific feed load (Equation (1)). Different maximum specific feed loads were observed for the three polymers. These differences were attributed to material properties and were subsequently investigated. In the second phase of the optimization procedure (Figure 4, C to D), the throughput was maximized at a constant maximum specific feed load. Therefore, the feed rate and screw speed were increased while the ratio between them remained constant. This procedure increased the melt temperature due to higher shear within the extrusion screw. In order to evaluate the melt temperature, autogenic extrusion (wherein the melt is neither heated nor cooled by the extrusion barrel) was used. The heat required for the elevated temperature of the melt was achieved by a conversion of the mechanical power of the screw to thermal energy at a given flow rate. This approach is preferred because it leads to a robust and scalable process [13]. The differences in the melt temperatures at similar mass flow rates were attributed to the different viscosities of the polymer melts, which were subsequently evaluated.

These experiments applied the SIOS for the first time to two other polymers (SOL and bBMA) which are commonly used in the hot melt extrusion of amorphous solid dispersions [24,25,26,27]. Autogenic extrusion was applied to the second phase of the SIOS for the first time, extending the concept to more robust operating conditions. Higher barrel loads and throughputs of up to 42 kg/h were achieved compared to previous investigations [14] (Figure 3, barrel 2). The upper process limit for the throughput was determined by the feeding system rather than by degassing or thermal degradation (coloring) of the polymer. 

### 3.2. Maximum Barrel Load

The specific feed load is a dimensionless number that represents the load of the extrusion barrel. Different maximum specific feed loads ($SFL_{max}$) were observed for the three polymers (SOL > bBMA > PVPVA), which are characterized by the horizontal line between points C and D (Figure 4). The ranking of the $SFL_{max}$ is the same order as bulk densities ($\rho_{bulk}$) (315 kg/m³ for PVPVA, 597 kg/m³ for SOL and 339 kg/m³ for bBMA). The definition of the SFL was adapted to further elucidate this effect. Generally, the SFL is the ratio between the material volume flow rate ($\dot{m}/\;\rho_{bulk}$) and the transport capacity of the extruder ($n\cdot d^{3}$). A backlog occurs when the transport capacity of the extruder is exceeded. However, the description of the transport capacity is quite poor and does not lead to a meaningful absolute value for the SFL, since screw geometry, as well as the transport behavior of a specific material, is not considered (Equation (1)). Therefore, the free cross-sectional area ($A_{free}=0.000491\;m²)$ and the pitch ($l_{pitch}$) of the screw (feeding section) were considered, as well as the slip (s) of the powder (Equation (5)). Since the backlog occurred in the feeding zone, where the material is in its bulk powdered state, the bulk density was used instead of the true density (1190 kg/m³ for PVPVA, 1080 kg/m³ for SOL and 1092 kg/m³ for bBMA). In this way, a normalized SFL value ($SFL^{*}$) can be obtained, where $SFL^{*}=0$ corresponds to an empty feeding section, and $SFL^{*}=1$ corresponds to a completely filled feeding section.

(5) $$SFL^{*}=\frac{\dot{m}/\;\rho_{bulk}}{\left(1-s\right)\cdot l_{pitch}\cdot A_{free}\cdot n}$$

Using this equation, the slip was calculated assuming filled barrels in the feeding zone ($SFL^{*}=1$). The values were quite similar (0.865 for PVPVA, 0.858 for SOL and 0.850 for bBMA), which indicated a comparable powder flow within the extrusion barrel. Therefore, the differences in $SFL_{max}$ are mainly attributed to differences in the bulk density. Using this approach, a model was developed to calculate the $SFL_{max}$ based on extruder geometry, bulk density and slip of the polymers (Equation (6)).

(6) $$SFL_{max}=\left(1-s\right)\cdot \frac{\rho_{bulk}}{\rho_{true}}\cdot \frac{A_{free}\cdot l_{pitch}}{d^{3}}$$

Based on this equation, the first step in the SIOS can be skipped (point A to B to C) in order to save time and resources by conducting preliminary experiments to determine the powder slip.

### 3.3. Melt Temperature

In the autogenic extrusion (Figure 4, C to D) different melt temperatures were observed for comparable throughputs using different polymers (Figure 4), which was related to the polymer’s melt rheology. In order to evaluate this further, the extruder was treated as a capillary rheometer in which the viscosity at the die ($\eta_{die}$) is related to shear stress ($\tau_{die}$) and shear rate (${\dot{\gamma }}_{die}$) in the die, and Newton’s law of viscosity applies (Equation (7)).

(7) $$\eta_{die}=\tau_{die}\cdot \frac{1}{{\dot{\gamma }}_{die}}$$

According to Hagen–Poiseuille law [22] (Equation (8)), the shear stress is a function of the pressure drop ($∆p_{die}$) across a cylindrical die with a radius ($r_{die}$) and length ($l_{die}$). The shear rate is related to the radius and volume flow rate though the die (${\dot{V}}_{die}$).

(8) $$\eta_{die}=\frac{∆p_{die\;}r_{die}}{2l_{die}}\cdot \frac{\pi r_{die}^{3}}{4{\dot{V}}_{die}}$$

However, the Hagen–Poiseuille law does not apply to shear thinning materials such as the used polymer melts due to the parabolic shear rate distribution in the die opening [22]. Therefore, the shear rate of the Hagen–Poiseuille law was corrected (${\dot{\gamma }}_{die}^{corr}$) according to Weissenberg-Rabinowitsch [28], using the flow index (n) from Ostwald law, which can be derived from the flow index of Carreau (c=1−n) (Equation (9)) [29].

(9) $${\dot{\gamma }}_{die}^{corr}=\frac{3n+1}{4n}\;{\dot{\gamma }}_{die}=\frac{4-3c}{4-4c}{\dot{\gamma }}_{die}$$

At this point, it is worth mentioning that the die shear stress ($\tau_{die}$) is systematically affected by shear rate due to imperfect laminar flow conditions at the entrance of the die. This has been studied by Bagley and Cogswell [30,31] and is the reason for the common twin-die setup of capillary rheometers or dual measurement protocols. This will be addressed subsequently but should be ignored for the moment to allow relative comparison of the polymers. 

**Figure 5:**
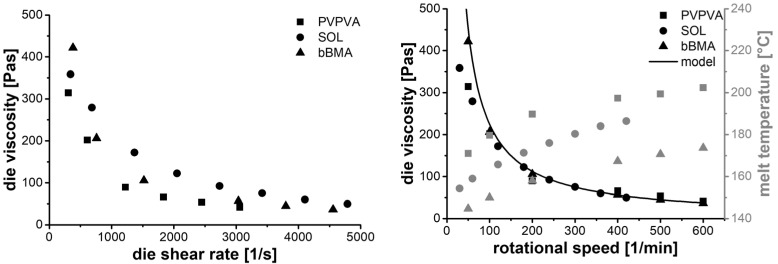
Results of autogenic extrusion experiments (Figure 4, C to D). Die viscosity as function of die shear rate (**left**), die viscosity (black) and melt temperature (grey) as function of screw speed (**right**).

The flow functions (Figure 5, left) show distinct differences between the polymers; materials with higher *SFL* values exhibit higher die viscosities at similar die shear rates. This is related to the lower screw speed and less shear at similar volume flow. The shape of these flow functions is unusual compared to the literature ([19,20], data not shown). However, in the twin-screw extrusion process, the shear rate is varied by the volume flow (Equation (8)), but the material temperature adapts automatically as well (Figure 4, C to D). In fact, each data point of Figure 5, left, was taken at a different melt temperature, which is the reason for the unusual shapes.

When considering the die viscosity as a function of screw speed (Figure 5, right), the die shear rate varies between the materials for constant screw speed due to different *SFL* values (Equation (1)). However, the shape of this function is quite similar for the three polymers, which was unexpected. Apparently, each screw speed of the extrusion screw leads to the same die viscosity, regardless of the material. Moreover, a hyperbolic trend was observed in the data and was used to model the data with Equation (10).

(10) $$\eta_{die}=\tau_{extruder}\cdot \frac{1}{n}$$

Here, n is the screw speed, which correlates to the shear rate in the extruder screw (compare Equation (7)). The correlation parameter ($\tau_{extruder}$) can be thought of as a characteristic shear stress within the extruder die, having a constant value for all screw speeds, since it establishes automatically. These shear stress values were quite similar for all three polymers (Table 2), so an average value was used to model the behavior in Figure 5, right. Only the data points at particularly low screw speeds (below 100 rpm) and low throughput are not well described by this model. The extruder shear stress appears to be a characteristic parameter specific to the type of extruder with a given screw configuration and die geometry. It should be suitable to transfer process conditions between materials, as the particular value has no physical meaning because the screw speed, and not a real shear rate, is used.

Apparently, the extruder produces a melt with a particular viscosity for a specific screw speed, independent of the material rheology. This observation can be explained based on the steady-state operation of the extruder. For high viscosities, more mechanical energy is transferred from the extruder screw to the material, which increases the temperature and lowers the viscosity. Low viscosities, on the other hand, lower the energy dissipation, which leads to less of an increase in the material temperature and a rise in the melt viscosity. That means that the screw speed alters the material temperature until the corresponding viscosity is obtained (Figure 5, right). Because of this, the die viscosity correlates much more with the screw speed than the die shear rate.

**Figure 6:**
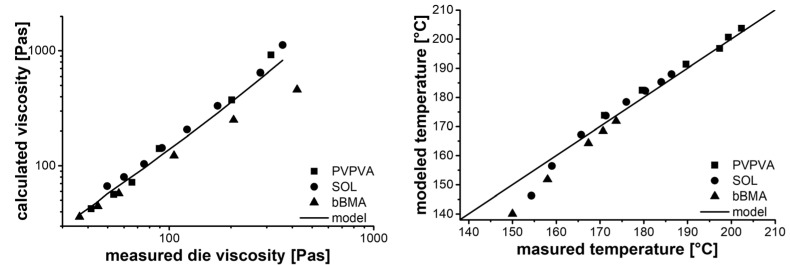
Results of autogenic extrusion experiments. Correlation between die viscosity and calculated viscosity (**left**), comparison of measured and predicted melt temperature (**right**).

Further investigations examined the correlation of the die viscosity with the viscosity from the Carreau–Arrhenius (calculated viscosity) model using die shear rate and melt temperature. Ideally, these two viscosities should be the same, but there are several limitations to using a production scale extruder as a capillary rheometer, such as the in-feed behavior until laminar flow is reached. However, a correlation between these two viscosities was found (Figure 6, left)—although it breaks down at high viscosities and low shear rates (low throughput). This phenomenon was not evaluated further since it only appeared below 10% of the nominal capacity of the extruder and high throughputs are usually desired in terms of process development. It is likely that at low throughput, the equilibration time was too low to establish autogenic conditions. Therefore, the model parameters (Table 2) were calculated from extrusion experiments with screw speeds of more than 100 rpm. At low viscosities (shear rates) the ratio between die viscosity and calculated viscosity (Carreau) converge to a value of about one. 

The two viscosities were correlated to the screw speed in a double logarithmic approach, which is quite common in the field of rheology [22].

(11) $$lg\frac{\eta_{die}}{\eta_{calculated}}=s_{extruder}\cdot lg\frac{n}{n_{max}}+i_{extruder}$$

In this way, the measured die viscosity ($\eta_{die}$) is normalized to the calculated viscosity by the Carreau–Arrhenius equation ($\eta_{calculated}$), while the screw speed of the screw (n) is normalized to the nominal speed ($n_{max}$) of the extruder. The slope ($s_{extruder}$) and the intercept ($i_{extruder}$) are extruder-specific parameters, including die geometry and screw configuration. These should be independent from process conditions and material properties. The individual parameters for the three polymer materials are given in Table 2, wherein a slight deviation of the bBMA from the other polymers can be seen. This might be related to imperfections in the extrusion process, issues with the rheological data from the literature and even batch-to-batch variability of the polymer. However, the origin remains unclear. 

Based on the two aforementioned correlations, it is possible to predict the die temperature based on the screw speed, since the screw speed can be used to calculate the die viscosity using Equation (10), and the die viscosity can be converted to an extruder-independent viscosity ($\eta_{calculated}$) by Equation (11). Knowing the rheological behavior of the material, the extruder-independent viscosity can be assigned to a resulting temperature using the die shear rate Equation (8), the Carreau Equation (3), as well as the Arrhenius approach (4). Unfortunately, the Carreau equation cannot be solved analytically for the shift factor (a) and the related temperature (T), due to the exponential nature of the flow index (c). Therefore, numerical solving methods were used. 

Comparing the measured and modeled temperatures (Figure 6 right), adequate agreement was found. Noticeable deviations were only observed for low throughput (low temperatures), as discussed before. 

### 3.4. Guidance for Application

Based on the previous results, the scale-independent optimization strategy was adapted to find an appropriate operating point (high throughput and desired melt temperature) with fewer experiments (SIOS 2.0). A stepwise procedure is presented as follows:

1. Determine the following material characteristics:
  - powder bulk density ($\rho_{bulk}$)
  - material density ($\rho_{true}$)
  - melt rheology (e.g., Carreau–Arrhenius)
2. Determine the following extruder parameters:
  - free cross-sectional area of the screw ($A_{free}$)
  - screw diameter ($d^{3}$)
  - screw pitch in the feeding section ($l_{pitch}$)
  - die radius ($r_{die}$)
  - die length ($l_{die}$)
3. Determine maximum mass flow (${\dot{m}}_{max}$) that is transported through the feeding section of the extruder using different screw speeds (*n*). Calculate the slip (s) from the slope of linear regression using Equation (12).
  (12) $${\dot{m}}_{max}=\left(1-s\right)\cdot l_{pitch}\cdot A_{free}\cdot \rho_{bulk}\cdot n$$
4. Investigate the extruder performance in autogenic conditions using at least two screw speeds at maximum specific feed load.
  - Set the barrel temperature to the manufacturer’s recommended process temperature for the material. Cool the feeding section to ambient temperature to avoid clogging.
  - Choose a reasonable screw speed (e.g., 200 rpm) and set the mass flow for this specific speed using Equation (5) at SFL* = 1. Note that low screw speeds increase the risk of clogging the extruder (exceeding torque limit), while high speeds increase the material consumption.
  - Wait for the steady state of the extrusion process at these conditions, at which point, homogeneous, coherent (commonly transparent) extrudate strands are obtained.
  - Turn off the barrel temperature control to allow autogenic extrusion. However, continue cooling the feeding section and heating the die to the manufacturer’s recommended process temperature for the material (particularly necessary for small extruders).
  - Measure the melt temperature (e.g., with an IR thermometer) after steady state is reached (power consumption, die pressure and the barrel temperature are constant), which may take several minutes. Note the screw speed, mass flow rate, melt temperature and die pressure.
  - Move to other process conditions. If the power consumption is reasonably low (less than 50% of the nominal value), the screw speed and the mass flow rate should be lowered by the same ratio (e.g., two-thirds). If the power consumption is relatively high, the higher screw speed and mass flow rate should be increased by the same ratio (e.g., four-thirds).
  - Measure the melt temperature and die pressure after reaching steady state. Note the corresponding mass flow rate and screw speed.
  - Fit the model parameters ($\tau_{extruder}$, $s_{extruder}$ and $i_{extruder}$) in Equations (10) and (11), which describe the correlation between screw speed and extruder-independent viscosity. Calculate the melt temperature as a function of screw speed (Section 3.3).

**Figure 7:**
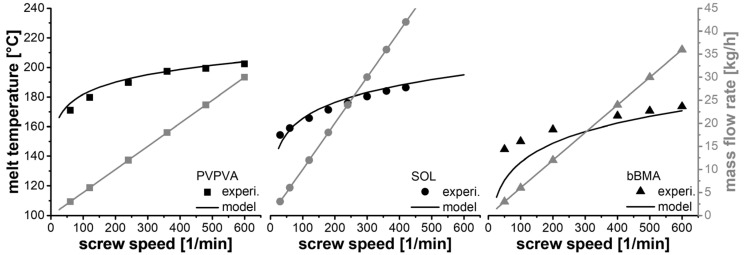
Comparison between modelled and experimental data considering melt temperature (black) and mass flow rate (grey). The extruder parameters for modelling one polymer were derived from the two other polymers.

The predictive power of this new concept was evaluated using the same experimental data that were presented before. However, the data set was reevaluated in a crossover design. The extrusion performance of one polymer was predicted by the two other polymers and subsequently compared to the experimental data (Figure 7). 

The melt temperatures of PVPVA and SOL were predicted well using the new model. There were systematic deviations at low screw speeds, which might be attributed to incomplete thermal equilibration. However, the desired range for any production process will be at a high speed and high throughput. The predicted melt temperature of bBMA is systematically lower than the measured values. The extrusion behavior of this polymer is less related to the rheological data found in the literature. However, temperature differences of 5K at the desired process conditions seem to be acceptable. The prediction of the mass flow rate reflects the differences in slip between the polymers. Since deviations were of less than two percent, no relevant differences between the model and experiment were recognized. 

These results demonstrate the predictive power of the new modelling concept SIOS 2.0. Direct process parameters, such as screw speed and mass flow rate, can be chosen, and dependent process parameters, such as specific feed load and melt temperature, can be predicted. Since this concept is valid for polymers with different structures, it is likely that this concept would be valid for more similar materials, such as polymers and their corresponding amorphous solid dispersion formulations.

## 4. Conclusions

In this work, the scale-independent optimization strategy (SIOS), according to Wesholowski et al. [14], was extended to include the autogenic extrusion mode and was applied successfully to two new polymers. 

Differences between the polymers in terms of the maximum barrel load and melt temperature were attributed to the critical material attributes. The maximum barrel load was found to be dependent on the polymer bulk density and the process-related slip. The melt temperature was related to polymer rheology, and it adjusted automatically based on the screw speed. Two mathematical models were developed to predict the throughput, as well as the melt temperature, based on extruder dimensions, material properties and several extrusion experiments. Thus, the existing SIOS was extended to SIOS 2.0, reducing the time and experimental effort.

## Figures and Tables

**Table 1:** Rheology parameters for the polymers based on a refit of literature data using Carreau–Arrhenius approach.

		Carreau		Arrhenius	
Substance	$\eta_{0}\;\left[\mathrm{Pa}\;s\right]$	${\dot{\gamma }}_{c}\;\left[1/s\right]$	c [−]	$E_{a}\;\left[J/\mathrm{mol}\right]$	$T_{Ref}$ **[K]**
PVPVA [19]	169.7	133.1	0.387	198,292	473
SOL [20]	147.3	136.7	0.411	150,773	473
bBMA [20]	25.58	1688	0.561	140,336	473

**Table 2:** Model parameters for autogenic extrusion (Equations (10) and (11)). Specific to a particular extruder in a certain setup (av ± s), independent form material and process parameters.

Substance	$\tau_{extruder}\;\left(Pa\right)$	$s_{extruder}\;\left(-\right)$	$i_{extruder\;}\left(-\right)$	R (−)
PVPVA	399 ± 26.9	0.409	0.131	0.958
SOL	361 ± 12.3	0.318	0.036	0.986
bBMA	361 ± 14.2	0.126	0.045	0.979

## Data Availability

The raw data supporting the conclusions of this article will be made available upon request.

## Appendix A

**Table A1:** Experimental data from Scale-Independent Optimization Strategy (no pressure data for the first phase—since no thermal equilibration).

Polymer	n (1/min)	$\dot{m}$ (kg/h)	T (°C)	p (MPa)
PVPVA	200	3	181.0	
	150	3	178.7	
	100	3	174.3	
	80	3	173.0	
	60	3	170.7	
	50	3	170.7	
	60	3	171.0	2.08
	120	6	179.7	2.51
	240	12	189.7	2.28
	360	18	197.3	2.46
	480	24	199.3	2.62
	600	30	202.3	2.56
SOL	200	3	168.3	
	150	3	166.0	
	100	3	163.3	
	80	3	160.7	
	60	3	158.3	
	40	3	156.0	
	20	3	154.3	
	30	3	154.3	2.49
	60	6	159.0	3.59
	120	12	165.7	4.26
	180	18	171.3	4.49
	240	24	176.0	4.51
	300	30	180.3	4.59
	360	36	184.0	4.42
	420	42	186.3	4.30
bBMA	200	3	152.3	
	150	3	150.7	
	100	3	147.3	
	80	3	146.3	
	60	3	145.7	
	40	3	144.0	
	50	3	145.3	
	50	3	144.7	2.50
	100	6	150.0	2.44
	200	12	158.0	2.50
	400	24	167.3	2.68
	500	30	170.7	2.65
	600	36	173.7	2.59
