# Correction: Gottschalk et al. Predicting Throughput and Melt Temperature in Pharmaceutical Hot Melt Extrusion. *Pharmaceutics* 2022, *14*, 1757

**DOI:** 10.3390/pharmaceutics17121523

**Published:** 2025-11-27

**Authors:** Tobias Gottschalk, Cihangir Özbay, Tim Feuerbach, Markus Thommes

**Affiliations:** 1 Laboratory of Solids Process Engineering, Department of Biochemical and Chemical Engineering, TU Dortmund University, Emil-Figge-Str. 68, 44227 Dortmund, Germany; 2 INVITE GmbH, Drug Delivery Innovation Center, Chempark Building W32, 51368 Leverkusen, Germany

## Appendix

In the original publication [1], there was a mistake in Table A1 as published. The pressure data given in the appendix for 2 of 3 substances also include the offset of the pressure sensor due to an editing error. Therefore, it is impossible for the reader to reproduce the result given in the manuscript based on the supplementary data. The authors suggest adding the sensor offset to the table. The corrected Table A1 appears below. The authors state that the scientific conclusions are unaffected. This correction was approved by the Academic Editor. The original publication has also been updated.

**Table A1:** Experimental data from Scale-Independent Optimization Strategy (no pressure data for the first phase—since no thermal equilibration).

Polymer	n (1/min)	$\dot{m}$ (kg/h)	T (°C)	p (MPa)
PVPVA	200	3	181.0	
	150	3	178.7	
	100	3	174.3	
	80	3	173.0	
	60	3	170.7	
	50	3	170.7	
	60	3	171.0	2.08–0.58
	120	6	179.7	2.51–0.58
	240	12	189.7	2.28–0.58
	360	18	197.3	2.46–0.58
	480	24	199.3	2.62–0.58
	600	30	202.3	2.56–0.58
SOL	200	3	168.3	
	150	3	166.0	
	100	3	163.3	
	80	3	160.7	
	60	3	158.3	
	40	3	156.0	
	20	3	154.3	
	30	3	154.3	2.49–0.58
	60	6	159.0	3.59–0.58
	120	12	165.7	4.26–0.58
	180	18	171.3	4.49–0.58
	240	24	176.0	4.51–0.58
	300	30	180.3	4.59–0.58
	360	36	184.0	4.42–0.58
	420	42	186.3	4.30–0.58
bBMA	200	3	152.3	
	150	3	150.7	
	100	3	147.3	
	80	3	146.3	
	60	3	145.7	
	40	3	144.0	
	50	3	145.3	
	50	3	144.7	2.50
	100	6	150.0	2.44
	200	12	158.0	2.50
	400	24	167.3	2.68
	500	30	170.7	2.65
	600	36	173.7	2.59
